# Editorial

**Published:** 1862-03

**Authors:** 


### Editorial.

American Medical Association: Further Postponement. —We, the undersigned Committee of Arrangements of the American Medical Association, after free consultation with officers and members in each important section of the country, accessible to the Committee, feel constrained to give notice to the profession, that the Regular Annual Meeting of the Association is further postponed until the first Tuesday in June, 1863.

Chicago, March 20, 1862.

N. S. Davis,

J. Bloodgood,

J. W. Freer,

H. W. Jones,

E. Andrews,

D. Laskie Miller,

Thomas Bevan.

} *Committee.*

We call the attention of our readers to the above notice.—In our last number, we expressed the hope that the Association would hold its regular meeting in June next. But out of quite a large number of letters received from influential members of the Association, all but *two* advised a further postponement. Of course we cheerfully acquiesce in the decision of the Committee, founded as it appears to be, on the wishes of a majority of the profession. We hope and trust that another year will find all the obstacles to a truly great *national* meeting removed, and we shall have the pleasure of again seeing our brethren of the profession from Maine to Texas, assembled in cordial, scientific and social communion.

---

Close of the Medical College Terms in this City—Prosperity of the Colleges—Prospects for the Future.—In the preceding number of the Examiner we announced the closing exercises of the Lind University, which took place on the 4th inst. The annual term in the Rush Medical College had closed a month earlier. The winter passed pleasantly, and so far as we know, satisfactorily, in both schools.

We are informed that the class in the Rush Medical College was about 120, and the number of graduates 36; which is about the average of annual attendance in that school for the last six years. The Medical Department of the University, which was organized only three years since, opened the first year with a class of 33 students, of whom 9 graduated.

The second year opened with 51 students, of whom 12 graduated; while the class for the term just closed, numbered 63 students, of whom 16 graduated. This not only shows a healthy and very satisfactory degree of prosperity on the part of the University School, but already places it alongside of the Albany and New York Medical Colleges; and quite in advance of the Medical Departments of Boudoin, Yale, and some of the older schools of the East. We are all the more gratified, that this prosperity, has not been accompanied by any corresponding diminution of the classes in the Rush Medical College. When, three years since, we took an active part in organizing the Medical Department of the University, some were ready to censure us, on the ground that it would only result in dividing the patronage, already too sparingly bestowed on the only School then in existence here. We then claimed that the organization of a new School, with a longer lecture term, a more numerous Faculty, a more systematically arranged, and much more comprehensive course of instruction, so far from injuring the Rush Medical College, would only serve to excite the Faculty to greater exertions, by which her classes would be retained, and perhaps increased; while the new School would attract hither an entirely new and different class of students, thereby simply *adding to*, instead of dividing the aggregate number of students annually educated in this metropolis of the North-West. Thus far our predictions have been fully verified in practical results. Only three years have passed, and already the aggregate number in attendance at the two Schools is nearly *double* that which was in attendance on the one before the second was organized. And what is equally important, the competition has caused a far more active development of all the collateral departments, and means of medical advancement, such as better means of illustration in all the branches, and especially more extensive demonstrations with the microscope in histology; by vivisections in physiology; more extensive clinical courses; and the addition of well organized summer instruction; all of which has redounded greatly to the advantage of the Medical Classes. We say then, to the profession of the North-west, that the cause of Medical teaching and education in Chicago, is upward and onward; and that students can find here in our Schools and Hospitals, and Dispensaries, as good facilities for obtaining a thorough medical education, as in any other city in the Union.

---

Death of Dr. J. N. Graham.—At a regular meeting of the Chicago Medical Society, Drs. N. S. Davis and D. D Waite were appointed a Committee to draft resolutions expressive of the feelings of the Society towards the deceased.

Dr. Graham had been a resident of the city eight or ten years, and so long as his health permitted, was an active and highly esteemed member of our local Medical Societies. He was a physician of more than ordinary intelligence; kind, charitable, and faithfully devoted to his profession. He was highly respected and beloved, not only as a physician, but as a Christian of deep piety, by a large circle of acquaintances and friends.

The Committee reported the following resolutions, which were unanimously adopted, viz:

*Resolved*, That in the death of JOSEPH N. GRAHAM, M. D., this Society has lost one of its most active and esteemed members; the profession one of its most zealous and faithful cultivators; and the community one of its most amiable, intelligent, and devoted professional servants.

*Resolved*, That we deeply sympathize with his bereaved family and more intimate circle of relations and friends.

*Resolved*, That the foregoing be published in the Medical Journals, and a copy transmitted to the family of Dr. GRAHAM.

---

THE COUNTRY PRACTITIONER.—From an *Annual Address*, by the late Dr. D. T. JONES, President of the New York State Medical Society, for 1861, we copy these extracts, concerning a class of whom he, himself, was a shining exemplar —a class, illy paid, over-worked and under-rated—the *Country Practitioner*:—

A French philosopher is reported to have said that, at the end of a century, the newspaper will be the only book possible. So far as medical literature is concerned, we are nearing that point very rapidly. Already do periodicals furnish the busy practitioner with the larger share of his professional reading; in them he finds a living panorama of the daily improvements in his chosen profession. Necessity, in the country, prompts to this. Your city physician is always near to help in any extremity—the best council, the best books, and time to search them. Does it ever occur to the inhabitants of our small towns and villages, of the extra importance to them of their family physician? Do they understand that, in more senses than one, he holds their thread of fate? You or some of your family, may be suddenly struck down, past all hope, but by a surgeon's skill. Then how important that he be equal to the emergency. He goes alone, unaided. Anxiously have they awaited his arrival. How searching the glance, that seems to look into the inmost soul, as they ask, "Doctor, can you save me?" How the blood rushes back to their pallid cheeks at his assuring answer, "I can."

My hearers of the laity, if any are present, should a young man come into your neighborhood to practice his profession of medicine and surgery, examine him well, weigh his claim to your confidence, especially as to education, sobriety, morality, and good common sense. If you find such an one among you, cherish and encourage him, remembering that every moment spent in his studies is for your advantage; the idea he gets to-day, may save to you a darling life, or prevent issuing, in your own case, the sentence, "this night thou shalt surely die."

It will cost you but little. His aspirations for this world's goods are limited, or he is destined to be disappointed. He will have little need to use the prayer of Agur, "Give me neither poverty nor riches," for country fees give a bare support, nothing more.

* * * They who write of the "currents and counter-currents of medicine" would seem to know but little of country practice, or country practitioners.

The man who is his own apothecary, who measures, weighs, compounds, and doses his own medicine, will seldom be found giving too much or too many kinds. His materia medica will be very simple, but very certain. His weapons are not shot guns, loaded and fired under his direction, but Minie rifles, aimed by no eye except his own. No ignorant or dishonest apothecary comes between him and his patient.

American Journal of Ophthalmology.—Dr. Julius Homberger has issued a prospectus of a new journal with the above-title, to be published in New York, bi-monthly, at $2 per annum. It is stated in the prospectus that Dr. H. was a pupil of De Graefe, was late assistant to Dr. Sichel, Paris, and is a member of the Société Universelle d'Ophthalmologie, etc. We wish him success in his new enterprise. L. W. Schmidt, 534 Broadway, above Spring Street, is to be the publisher.

Temperature and Ventilation.—The Paris *Siecle says* that, generally speaking, during winter, apartments are too much heated. The temperature in them ought not to exceed fifteen degrees centigrade, (fifty-nine degrees Fahrenheit,) and even in periods of great cold, scientific men declare that twelve or fourteen degrees had better not be exceeded. In the wards of hospitals, and in the chambers of the sick, care is taken not to have greater heat than fifteen degrees. Clerks in offices, and other persons of sedentary habits, when the rooms in which they sit are too much heated, are liable to cerebral congestion and pulmonary complaints. In bedrooms, and particularly those of children, the temperature ought to be maintained rather low; it is even prudent only rarely to make fires in them, especially during the night. In addition to keeping up only a moderate temperature, the windows of all rooms, whatever the weather, ought to be opened for a time every day, so as to renew the air.

Food in Typhoid Fever.—Dr. Hérald confirms the opinion expressed some time ago on this continent as well as in England, that in the management of typhoid fever the all-important, the capital question is that of food; prolonged abstinence leads to the most disastrous results. One lady patient was sinking steadily under a strictly enforced abstinence; when the pulse numbered 120, nocturnal agitation with wandering delirium, vomiting and diarrhœa had set in, the effects of nutriment were tried. At first, only a few drops of iced beef-tea could be swallowed with the utmost difficulty; by and by the food remained on the stomach, and in proportion to its increase, the pulse fell, the delirium yielded and the patient recovered. Mr. Marotte has established that vomiting, diarrhœa and delirium, more especially the latter, are characteristic of starvation. Trouseau also lately pointed out the striking analogy existing between the more serious symptoms of typhoid fever and those of autophagy consequent upon protracted abstinence—*Berkshire Med. Journ.*, *from Championnière's Jourual.*

Sufferings of the Wounded at Fort Donelson.—The physical suffering of all who were engaged in the attack on and capture of Fort Donelson probably exceeded that of any previous battle on the continent. The temperature was lower than on the occasion of the famous winter fight at Eylau, and the misery of the men, from wet and frozen clothing, and insufficient food, was perhaps as great. On Thursday, the first day of the fight, the temperature was but ten degrees Fahrenheit, and snow fell during the day and the succeeding night. Feet and hands were, in many instances, frozen. It is believed that many of the wounded died where they fell from freezing, as the armies were so near together that neither belligerent could venture out to relieve the wounded or remove the dead. It is said that some of the wounded laid in the field from Friday until late on Sunday, and when rescued, were stiff with cold and covered with snow.

